# The Structure, Microhardness and Solid Particle Erosion Wear Characteristics of the Laser-Cladded Inconel 625 Coatings Reinforced with Cr_3_C_2_ Particles on Non-Alloy Steel Substrate

**DOI:** 10.3390/ma19132748

**Published:** 2026-06-27

**Authors:** Jacek Górka, Aleksandra Lont, Tomasz Poloczek, Marcin Żuk

**Affiliations:** Welding Department, Faculty of Mechanical Engineering, Silesian University of Technology, Konarskiego Street 18A, 44-100 Gliwice, Poland; tomasz.poloczek@polsl.pl (T.P.); marcin.zuk@polsl.pl (M.Ż.)

**Keywords:** laser cladding, Inconel 625, composite coatings, chromium carbide

## Abstract

The article presents the results of research concerning the laser cladding process of the Inconel 625 coatings reinforced with Cr_3_C_2_ particles. Nickel-based superalloys find widespread application in aggressive corrosive environments because of their many favorable properties, such as high tensile and fatigue strength and excellent resistance to high-temperature corrosion. The current research aimed to improve the surface wear resistance of Inconel 625 coatings by the addition of 20–40 vol.% of Cr_3_C_2_ particles. The research focused on determining the impact of the laser beam power and Cr_3_C_2_ particle content on the quality, structure and properties (microhardness and solid particle erosion wear resistance) of coatings. The investigation included the macrostructure and microstructure analyses, Energy Dispersive Spectroscopy (EDS), X-Ray Diffraction (XRD), Vickers microhardness and solid particle erosion tests (according to the ASTM G76-18 standard). The main results showed that the homogenous composite Inconel 625 + Cr_3_C_2_ coatings can be produced using the laser cladding process to improve the hardness and erosive wear resistance in comparison to pure metallic Inconel 625 coatings.

## 1. Introduction

Laser cladding is one of the surface treatment techniques designed to extend the service life of machine parts by applying a protective coating with a composition distinct from the substrate material while minimizing the negative heat impact on the structure, properties and thermal distortion of the substrate. The extensive use of the laser cladding technology across a variety of materials and applications is due to its many advantages, including the use of a highly concentrated heat source to fabricate thin coatings that are metallurgically bonded with the substrate, with a minimal heat-affected zone and distortion of the base material. Furthermore, the rapid cooling rates result in the formation of a unique, fine-grained microstructure with superior mechanical properties. Optimizing the process parameters allows control of the penetration and dilution of the substrate, coating thickness, surface roughness and the microstructure and properties by changing the heating and cooling rates. The automation of laser cladding ensures high precision and repeatability, making it a robust industrial solution [1,2,3]. Extensive research has demonstrated that this technology can be successfully used to produce a diverse range of coating compositions, most notably Metal Matrix Composites (MMCs) reinforced with ceramic particles to enhance wear resistance [4,5,6,7]. Specifically, coatings reinforced with oxides (Al_2_O_3_, ZrO_2_, CeO_2_, etc.), carbides (WC, TiC, SiC, Cr_3_C_2_, etc.) and nitrides (BN, TiN, AlN, etc.) have shown significant improvements in surface performance [8,9,10,11,12,13,14,15,16]. For example, Hu et al. [17] demonstrated the positive impact of a spherical WC particle addition on the surface wear resistance of Ni-based high-speed laser-cladded coatings. Tong et al. [18] improved the cavitation-erosion resistance of Ti6Al4V by in situ synthesis of TiC particles using laser cladding technology. Kyekyere et al. [19] applied laser cladding technology to improve the wear resistance of 16MnCr5 alloy by the addition of TiC and SiC particles.

Among the many different alloys used for protective coatings, nickel-based alloys and superalloys represent a critical group. Inconel 625, in particular, is frequently used due to its high-temperature oxidation and corrosion resistance in aggressive environments and high mechanical and fatigue strength. Consequently, it is extensively utilized in many industries, including aerospace, chemical, petrochemical and power generation [20]. However, for applications where, besides corrosion, wear mechanisms occur during service, Inconel 625 alloy does not exhibit high resistance. For such applications, MMC coatings are frequently used to combine the beneficial properties of the Inconel 625 alloy with enhanced surface wear resistance [21]. Such coatings may be fabricated using various methods, such as thermal spraying (e.g., plasma spraying, cold spraying, high velocity oxygen fuel spraying HVOF)), cladding (e.g., gas tungsten arc cladding, plasma cladding, laser cladding), or electroplating, utilizing various reinforcing particles (e.g., WC, VC, TiC, Cr_3_C_2_, TiB_2_) [22,23,24]. Considering the excellent performance of Inconel 625 at elevated temperatures, it is crucial to select reinforcing particles with high thermal stability, such as Cr_3_C_2_ carbides. Similar to the Inconel 625 alloy itself, these carbides are characterized by high resistance to oxidation and corrosion (including high-temperature corrosion), and their high hardness significantly improves the wear resistance of the surface [25]. The beneficial impact of the Cr_3_C_2_ reinforcement on the Ni-based coatings’ structure and properties was confirmed in numerous studies, but mainly with the use of thermal spraying methods [26,27,28,29,30,31,32,33,34,35,36]. Sidhu et al. [28] reported hot corrosion test results of HVOF-sprayed NiCr alloy coatings reinforced with 75% Cr_3_C_2_ particles. Roy et al. [29] investigated the structure and hardness of HVOF-sprayed Ni20Cr coatings with 75% Cr_3_C_2_. Verdi et al. [30,31] studied Inconel 625 + 20% Cr_3_C_2_ laser-cladded coatings, focusing on their microstructure and local wear behavior. Yang et al. [32] analysed the microstructure, microhardness and high-temperature dry sliding wear behavior of laser-cladded NiCr + 70% Cr_3_C_2_ and NiCr + 49 Cr_3_C_2_ + 30% WS_2_ coatings. Cao et al. [33] used laser cladding to fabricate Ni-based coatings reinforced with Cr_3_C_2_ particles and studied the influence of the NiCr intermediate layer on the structure, mechanical and tribological properties of the coatings. Lou et al. [34] reported the results of a study on the laser cladding parameters’ impact on the in situ fabricated NiCr + Cr_3_C_2_ coatings’ microstructure, microhardness, wear and corrosion resistance. Su et al. [35] also investigated the influence of the laser cladding process parameters on the Ni60A + Cr_3_C_2_ coatings using numerical analysis of the temperature field, phase composition analysis and tribological tests. Hebbale et al. [36] compared the laser cladding and microwave cladding on the microstructure, microhardness and wear behavior of NiCr + 75% Cr_3_C_2_ composite coatings.

Based on the previous studies in that field, there is a lack of information and research regarding the influence of chromium carbide reinforcement on the Inconel 625 solid particle erosion resistance. This study aims to investigate the influence of laser cladding parameters on the structure and properties of Inconel 625 alloy coatings reinforced with 20 vol.% and 40 vol.% Cr_3_C_2_ particles. The research conducted included macroscopic and microscopic observations, together with Energy Dispersive Spectroscopy (EDS) chemical composition analysis and X-Ray Diffraction (XRD) phase composition analysis, microhardness measurements and erosive wear resistance tests according to the ASTM G76-18 standard [37].

## 2. Materials and Methods

### 2.1. Materials and Laser Processing

The laser-cladded coatings were fabricated on the S355JR steel substrates (Table 1) (Cognor, Stalowa Wola, Poland) having dimensions of 100 mm × 100 mm × 10 mm. Before the laser processing, the substrate surface was prepared by grinding to surface finish of 0.5 μm Ra and degreasing with ethyl alcohol. For the laser cladding process, the Inconel 625 (Metcoclad 625, Oerlikon, Westbury, NY, USA, gas atomized spheroidal powder, Table 1) and Cr_3_C_2_ (Princeton Powder, New York, NY, USA) powders with the particle size range of 45 to 90 μm and 45 to 75 μm, respectively, were used. For this study, pure Inconel 625 powder and Inconel 625–Cr_3_C_2_ composite powder mixtures (at volume ratios of 80:20 and 60:40) were prepared prior to laser cladding process by mixing and drying at a temperature of 50 °C for 1 h.

Multi-run coatings with 40% overlap were produced using laser cladding with a TRUMPF Trudisk 3302 (TRUMPF, Ditzingen, Germany) solid-state laser setup (Table 2) equipped with a numerically controlled positioning system and a gravitational powder feeder. A 200 μm diameter laser beam focus was set 30 mm above the substrate surface. A geometry-optimized nozzle injected the powder directly into the molten pool. Argon was used for powder transport (3 L/min) and shielding (25 L/min). The entire process was conducted without preheating, ensuring an interpass temperature below 30 °C. Table 3 summarizes the specific cladding parameters.

### 2.2. Structure and Properties Testing

To assess the coating quality, liquid penetrant testing was performed using a color contrast technique. The inspection utilized 68 NF penetrant, MR 70 developer and MR 79 cleaner (MR Chemie, Unna, Germany). Both the penetrant dwell time and the development time were set to 10 min.

To analyze the structure of metallic and composite coatings, macrostructural observations were carried out using an Olympus SZX9 optical stereoscopic microscope (Olympus, Tokyo, Japan). Microstructural observations were performed with the use of an optical microscope, Nikon Eclipse MA100 (Nikon, Tokyo, Japan), and scanning electron microscope (SEM), ZEISS EVO MA10 (ZEISS, Jena, Germany), fitted with Bruker Energy-Dispersive Spectroscopy (EDS) analysis system (Bruker, Billerica, MA, USA). The metallographic samples were etched with aqua regia at 60 °C. To complete the macrostructural investigation, the geometric dimensions of the coatings (F_BM_—the cross-sectional area of melted substrate; RA—the cross-sectional area of coating) were measured using AutoCAD 2023 software (Autodesk, San Francisco, CA, USA) to calculate the dilution using Equation (1). The AutoCAD 2023 software was also used to calculate the pore surface fractions and diameters.

(1) $$U=\frac{F_{BM}}{F_{BM}+RA}\times 100[\%]$$

Additionally, to supplement the microstructural analysis, X-Ray Diffraction (XRD) analysis was carried out using a PANalytical X’Pert PRO (Malvern Panalytical, Malvern, UK) diffraction system equipped with a cobalt anode. The diffraction patterns were recorded in a continuous scan mode within the 2θ range of 30° to 125°, a step size of 0.0263° and a counting time per step of 17.34 s.

To evaluate the metallic and composite coatings, Vickers microhardness and solid particle erosion tests were performed. Microhardness was measured using a Future Tech FM-810 (FUTURE-TECH CORP., Kawasaki, Japan) Vickers microindentation tester at a 200 g load and 10 s dwell time. Measurements were done in two profiles: a vertical from the surface to the substrate (0.1 mm step) and a horizontal across the coating beads, 0.5 mm beneath the surface (0.5 mm step). The average microhardness and standard deviations were calculated based on the 24 individual results for each of the coatings (horizontal profile).

The solid particle erosive wear resistance was tested in accordance with the ASTM G76-18 standard [37]. As the erodent material, the 50 μm diameter angular Al_2_O_3_ abrasive particles in a stream of dry air were used. The tests were performed using an erodent velocity of 30 m/s and an abrasive particle feed rate of 2 g/min. The erodent was directed onto the surface for 10 min through a 1.5 diameter nozzle positioned 10 mm above the sample. The experiments were conducted at impingement angles of 90° and 30°, performing three replicates for each condition. The steady-state erosion rate (2) and erosion value (3) were determined according to ASTM G76-18 standard [37]. For this purpose, sample mass loss and coating densities were measured using a RADWAG AS 82/220.R2 PLUS (RADWAG, Radom, Poland) laboratory scale with an accuracy of 0.0001 g. The results for each of the tested coatings and impingement angles were additionally analysed using two-way ANOVA using Microsoft Excel. To determine the erosion mechanisms of the tested coatings, the SEM observations of the worn surfaces and the topography mapping using Leica DVM6 (Leica Microsystems, Wetzlar, Germany) digital microscope were carried out.

(2) $$Steady-state\;erosion\;rate\left[\frac{mg}{min}\right]=\frac{Mass\;loss\;\left[mg\right]}{Test\;time\;\left[min\right]}$$

(3) $$Erosion\;value\left[\frac{{mm}^{3}}{g}\right]=\frac{Volume\;loss\;\left[{mm}^{3}\right]}{Total\;mass\;of\;abrasive\;particles\;\left[g\right]}$$

## 3. Results and Discussion

### 3.1. Visual Inspection and Penetrant Testing

**Figure 1:**
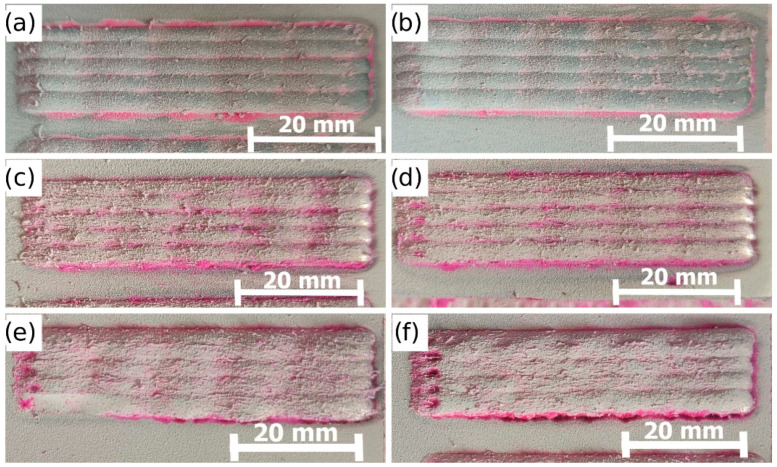
Coatings surfaces view after penetrant tests: (**a**) I1, (**b**) I2, (**c**) C1-1, (**d**) C1-2, (**e**) C2-1, (**f**) C2-2, designations according to Table 3.

Visual inspection of the coating surfaces and liquid penetrant testing (Figure 1) revealed no defects or indications on all of the analysed samples. Such results suggest the high quality of laser-cladded metallic and composite coatings using the selected process parameters.

### 3.2. Macrostructural Analysis

**Figure 2:**
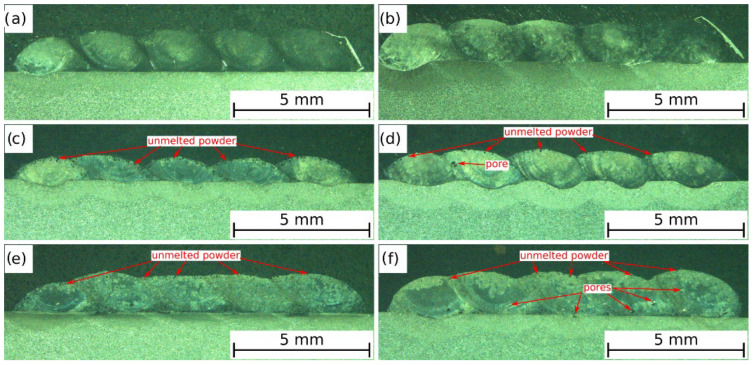
The macrographs of laser-cladded coatings: (**a**) I1, (**b**) I2, (**c**) C1-1, (**d**) C1-2, (**e**) C2-1, (**f**) C2-2, designations according to Table 3.

The macrographs of the laser-cladded coatings are presented in Figure 2, while the thickness and dilution measurements are summarized in Table 4. The macrostructural analysis aimed to assess the coatings’ quality and determine the influence of the laser beam power and powder composition on the geometry, penetration and dilution of the laser-cladded coatings. The macrostructural observations confirmed the surface visual and penetrant testing results: all of the coatings are crack-free. For all of the used powder compositions, lower laser beam power (and linear energy input) resulted in reduced penetration and dilution. For the constant laser processing parameters, the increased Cr_3_C_2_ content also caused a decrease in penetration and dilution, which is related to the Marangoni convection inhibition due to the carbide content increase in the used powder. It was previously proven [38,39,40] that dissolved carbon affects the molten pool surface tension gradient and can reverse the flow direction. In general, minimizing penetration is crucial for maintaining the chemical purity of the coatings by limiting dilution with the substrate material. However, sufficient metallurgical bonding must be achieved to ensure a defect-free interface, specifically preventing lack of fusion between the phases. At the selected parameters, even for higher laser beam power, the dilution remained minimal (up to approximately 10%). However, the slightly higher penetration depth at higher laser beam power reduced the risk of interface defects (which may locally occur in coatings I1 and C2-1). For all of the samples, increasing the laser beam power led to a slight increase in coating thickness. The macroscopic observations also revealed some pores trapped within the composite coatings clad at higher laser beam power, as well as areas of unmelted powder on the surface. The pore surface fractions for C1-2 and C2-2 were about 0.26% and 0.85%, respectively, while the average pore diameter was about 0.23 mm and 0.12 mm, respectively. Neither the porosity nor the unmelted powder areas were observed in the metallic Inconel 625 coatings fabricated under the same conditions. Porosity in the composite coatings may be attributed to the dissolution of Cr_3_C_2_ particles and the subsequent reaction of free carbon with the air, leading to the formation of CO and CO_2_ insoluble in the molten metal pool, which was previously described in [41,42]. Lower laser beam power and lower process energy may limit the dissolution of chromium carbides, so the pores were not found in those coatings. Additionally, the effect of Cr_3_C_2_ particle addition on Marangoni convection should also be considered, as the results show a slightly higher pore fraction for higher Cr_3_C_2_ volume content in the composite coatings. Similarly to the decreased dilution and penetration at higher Cr_3_C_2_ contents, such a result is caused by the Marangoni convection inhibition due to the higher carbon content, which can make the liquid metal pool degassing more difficult [38,39,40].

### 3.3. Microstructural Analysis

**Figure 3:**
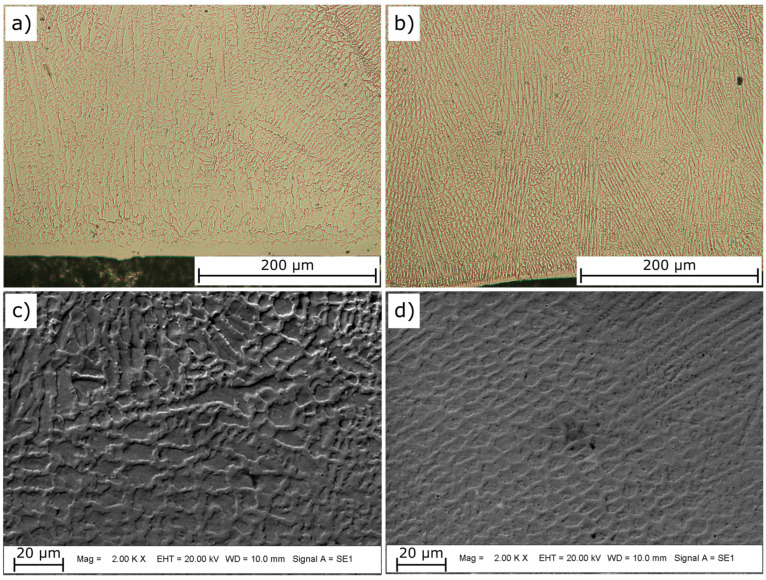
The microstructure of pure Inconel 625 coatings: (**a**) fusion line coating I1, (**b**) fusion line coating I2, (**c**) I2 coating, (**d**) I2 coating interpass area, designations according to Table 3.

**Figure 4:**
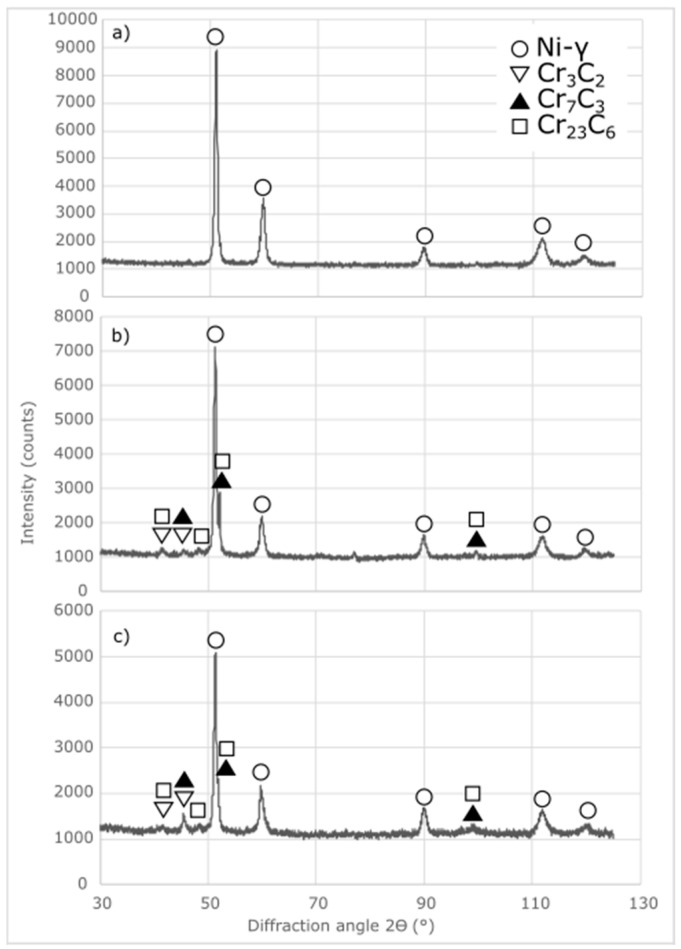
XRD patterns of coatings: (**a**) I2, (**b**) C1-2, (**c**) C2-2, designations according to Table 3.

The microstructures of the metallic Inconel 625 coatings are presented in Figure 3. These were analysed to assess the influence of Cr_3_C_2_ particles on the microstructural evolution. The metallic coatings exhibit a typical dendritic microstructure with minor constituents in the interdimeric regions, as previously described by Cieslak et al. [43,44]. XRD analysis (Figure 4a) confirmed that the metallic coatings consist of an austenite matrix; however, minor phases were not detected due to their low volume fractions in the coatings. At the coating–substrate interface, observations revealed the coarse planar and columnar grains, resulting from the high temperature gradient and relatively low solidification rate. The growth of the columnar crystals occurred in a direction consistent with the direction of heat transfer to the substrate material. In the upper regions of the metallic coatings, the typical variations in structural morphology and grain size were observed, driven by changing local solidification conditions. In the top area (close to the coating surface), the coaxial dendrites were observed, and slightly lower, both columnar and coaxial dendrites were observed. The primary dendrite arm spacing of columnar dendrites was measured close to the interface with the substrate material and in the upper coating areas. For the lower linear energy input, the average dendrite arm spacing was 1.95 µm and 4.05 µm in the lower and upper coating areas, respectively, while for higher laser beam power and energy input, it was 3.76 µm and 5,83 µm, respectively. These results show typical grain growth caused by the increased heat input between the two analysed coatings and the increased grain size in the upper areas of both coatings compared with the interface areas due to the lower temperature gradient and solidification rate. These results are consistent with previous studies [45,46].

**Figure 5:**
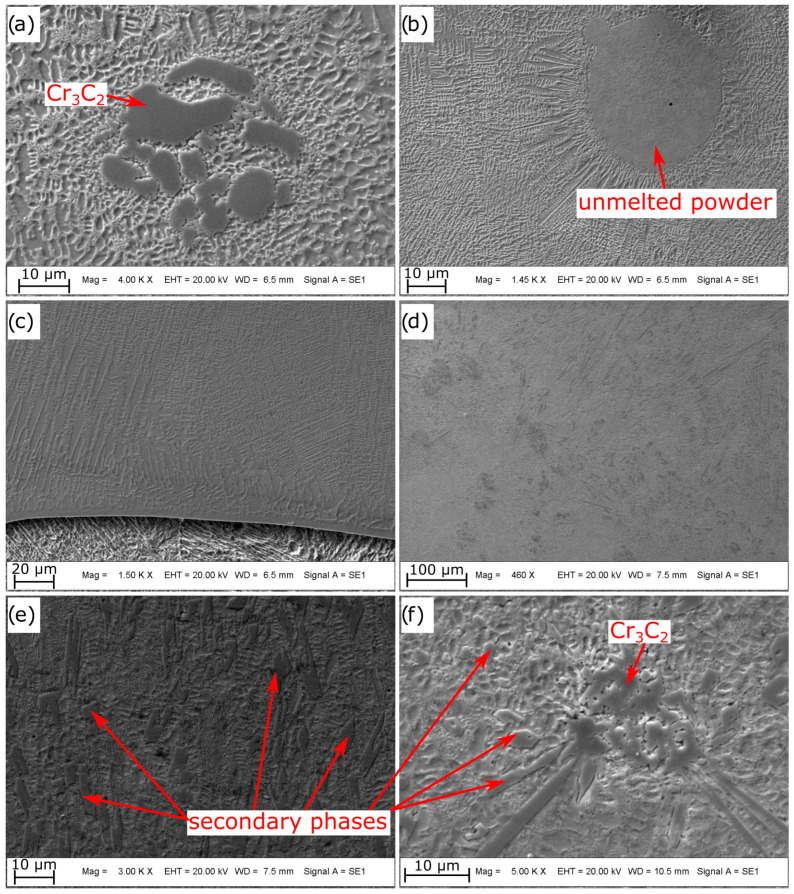
The microstructure of Inconel 625 + Cr_3_C_2_ coatings: (**a**–**c**) 20 vol.% Cr_3_C_2_, (**d**–**f**) 40 vol.% Cr_3_C_2_.

**Figure 6:**
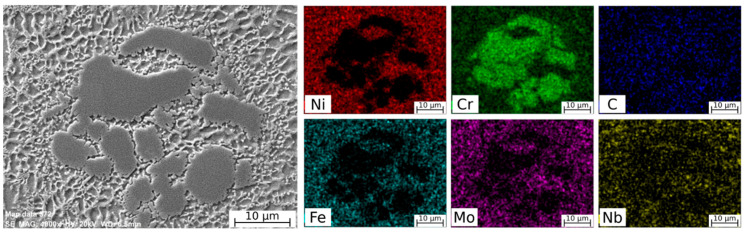
EDS mapping of a part of Inconel 625 + Cr_3_C_2_ coating C1-2, designation according to Table 3.

The microstructures of the Inconel 625 + Cr_3_C_2_ composite coatings are shown in Figure 5, while the EDS mapping of a Cr_3_C_2_ particle within the matrix is presented in Figure 6. Figure 4 shows the XRD analysis results for both the metallic and composite coatings. The composite coatings are composed of the austenite dendrites in the matrix (confirmed by XRD analysis), rich in Ni, Cr, Mo, Nb and Fe, along with ceramic reinforcing particles and secondary phases. Similar to the metallic coatings, coarse planar and columnar crystals were observed at the coating–substrate interface, where columnar crystals grew in the direction consistent with the heat transfer direction. The average primary dendrite arm spacing in this area was slightly higher than for the pure metallic coatings and increased with the higher Cr_3_C_2_ fraction (C1-1: 2.6 µm, C1-2: 4.12 µm, C2-1: 3.12 µm and C2-2: 4.26 µm). Such results may be attributed to the higher laser radiation level of the powder with higher carbide content, as the chromium carbides (Cr_3_C_2_) show higher optical absorption at the 1030 nm wavelength than nickel [47,48]. In the middle and upper regions of the coatings, the matrix morphology changed compared with the metallic coatings. The addition of ceramic particles promoted heterogeneous nucleation of the primary austenite dendrites (the observations show that dendrites initiate from the Cr_3_C_2_ particles). Such structural formation contributed to grain refinement. Although the dendrite arm spacing at the coating–substrate interface was slightly higher for composite coatings in comparison to metallic coatings, in the upper areas, the measured values were lower compared with the pure Inconel 625 coatings. The average primary dendrite arm spacing are 1.01 µm, 2.11 µm, 0.93 µm and 1.68 µm for C1-1, C1-2, C2-1 and C2-2, respectively. Similarly to the metallic coatings, in the case of composite coatings, the higher laser beam power and linear energy input caused increased grain size. An increase in the volume fraction of the reinforcing phase led to a reduction in the grain size in the analysed areas. Near the coating surface, where unmelted powder was observed (Figure 5b), these particles also acted as the crystal nucleus for the austenite grains. The Cr_3_C_2_ particles were properly wetted by the liquid metal, and no defects were found at the interface between the reinforcing particles and the matrix. Observations also confirmed a uniform distribution of the reinforcing particles throughout the composite coatings. However, the chromium carbide particles showed rounded edges, which suggests the partial dissolution of the reinforcing phase in the liquid metal pool, causing its enrichment with chromium and carbon. This dissolution led to the in situ formation of secondary phases during crystallization, which, according to the XRD analysis results (Figure 4b,c), may be Cr_7_C_3_ and Cr_23_C_6_ chromium carbides.

### 3.4. Microhardness and Solid Particle Erosion

**Figure 7:**
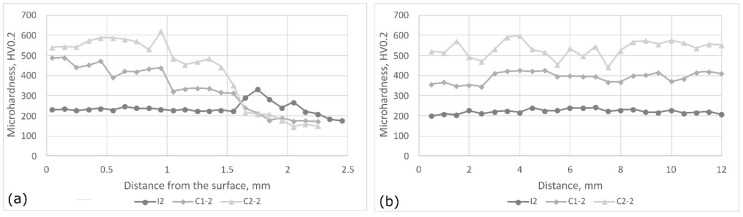
Microhardness distributions of coatings: (**a**) vertical profile, (**b**) horizontal profile, designations according to Table 3.

The average microhardness results are listed in Table 5, and the microhardness distribution profiles of representative coatings are shown in Figure 7. The selected laser cladding parameters did not significantly affect the average microhardness of the pure Inconel 625 coatings, which was approximately 222–225 HV0.2. The addition of 20 and 40 vol.% Cr_3_C_2_ to the Inconel 625 powder led to an increase in the average microhardness up to 427 HV0.2 and 533 HV0.2, respectively. The average microhardness results do not show a clear correlation with the measured coating dilution. For the pure metallic and composite coatings reinforced with 20 vol.% of Cr_3_C_2_, lower dilution with substrate was associated with higher average hardness; however, for the coatings containing 40 vol.% of Cr_3_C_2_, this trend was reversed. In general, the microhardness distributions across the coatings (both horizontal and vertical profiles) were highly uniform, particularly for the pure metallic coatings due to their more homogeneous microstructure. The measured microhardness of the Cr_3_C_2_ particles was approximately 1170 HV0.2. The presence of such hard particles within the Inconel 625 matrix caused higher local microhardness fluctuations, which were the most noticeable for the coatings reinforced with 40 vol.% Cr_3_C_2_ particles and impacted higher standard deviations for composite coatings in comparison to the metallic coatings. For the composite coatings, a slight decrease in microhardness was observed near the coating–substrate interface, resulting from dilution with the softer base material and lower Cr_3_C_2_ fraction in this region, as was observed during microstructural analysis. Analysis of the microhardness profiles indicates that significant hardening in the heat-affected zone (HAZ) did not occur, suggesting that the process parameters did not negatively impact the substrate’s properties. However, for the metallic coatings, some hardening in the HAZ was observed, likely due to different solidification conditions caused by variations in powder composition and laser beam absorption. No significant microhardness changes were detected at the interfaces between consecutive beads, confirming the high homogeneity of the multi-run coatings.

The solid particle erosion was tested for the I2, C1-2 and C2-2 coatings due to better quality (lower risk of the lack of fusion at the coating–substrate interface). The average steady-state erosion rates and erosion values for the tested coatings are summarized in Table 6. Table 7 presents the results of the two-way ANOVA for the steady-state erosion rate and erosion value, with coating type and impingement angle as factors. For each coating, the erosion rates and erosion values were higher for the impingement angle of 30° than 90°, which is characteristic of the materials exhibiting a ductile mechanism of erosive wear [49,50]. The two-way ANOVA results also confirm that the impingement angle highly impacts the solid particle erosion resistance. No significant variations in the measured values were observed for each of the coatings for the 90° impingement angle, while slight variations were recorded for the 30° impingement angle. Under this condition, an increased fraction of Cr_3_C_2_ particles resulted in lower steady-state erosion rates and erosion values, meaning that the solid particle erosion resistance improved. The two-way ANOVA interaction results (coating × impingement angle) confirmed that the change of the impingement angle caused similar trends in the erosion test results for all tested coatings. Moreover, the main effects of ANOVA indicated that the coating chemical composition (chromium carbide content) impacted the solid particle erosion wear resistance.

**Figure 8:**
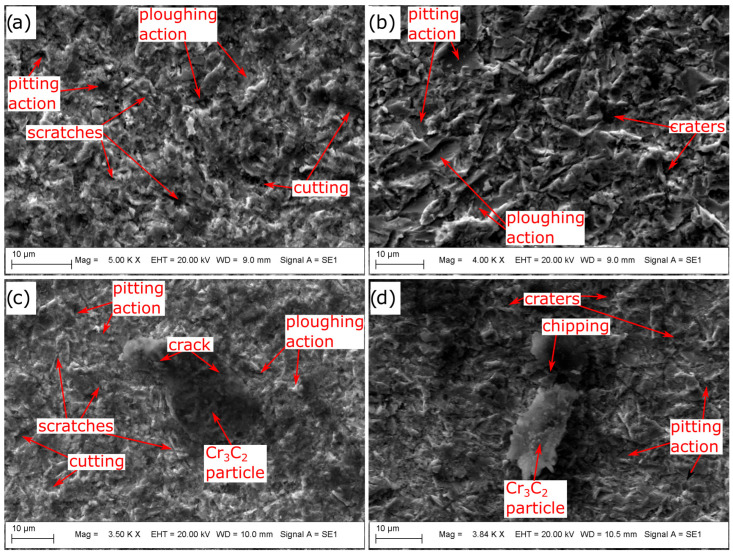
SEM micrographs of craters after solid particle erosion tests: (**a**) I2, 30°, (**b**) I2, 90°, (**c**) C2-2, 30°, (**d**) C2-2, 90°, designations according to Table 3.

**Figure 9:**
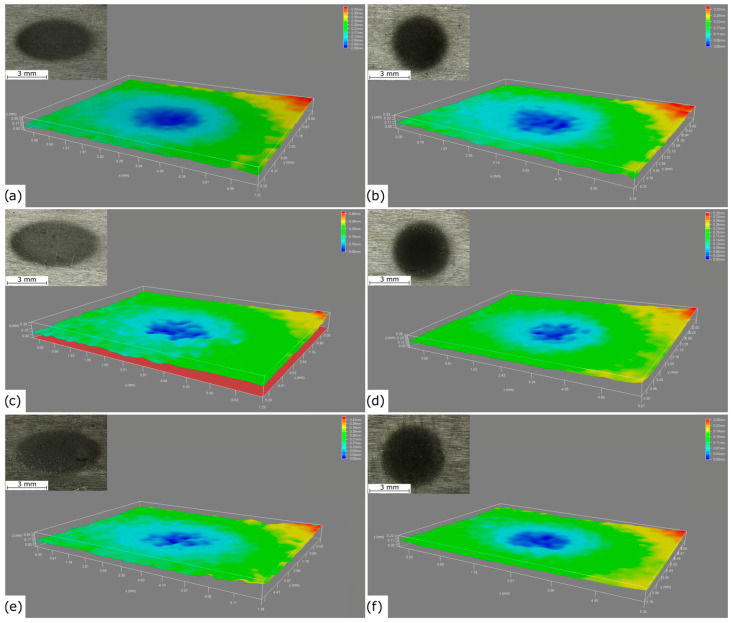
Topography maps of the worn surfaces after solid particle erosion tests: (**a**) I2, 30°, (**b**) I2, 90°, (**c**) C1-2, 30°, (**d**) C1-2, 90°, (**e**) C2-2, 30°, (**f**) C2-2, 90°, designations according to Table 3.

To analyse the erosion wear mechanism, SEM observations of worn surfaces (Figure 8) were combined with topography mapping using an optical microscope (Figure 9). The results demonstrate that the difference between the metallic and composite coatings’ microstructures affected the type of damage during solid particle erosion tests. Furthermore, the impingement angle also influenced the damage types and erosion mechanisms. For the pure metallic Inconel 625 coating, at both tested impingement angles, on the worn surfaces, the plastic deformation occurred without any evidence of cracking. At a 30° impingement, angle the material degradation occurred by scratching, micro-ploughing, micro-cutting and pitting action, while the craters, micro-ploughing and pitting damage were observed for the 90° impingement angle. For both angles, material removal was uniform across the entire tested area, which results from the high homogeneity of the microstructure and uniform properties of the metallic coatings, causing relatively smooth topographies of the worn surfaces. Such observations confirm the ductile mechanism of erosion wear of the Inconel 625 coatings. In contrast, the composite coatings exhibited a distinct difference in the wear behavior between the matrix and the reinforcing particles. The matrix material underwent plastic deformation similar to the pure metallic coatings, showing scratches, micro-cutting, micro-ploughing, pitting and craters depending on the impingement angle. Conversely, the Cr_3_C_2_ reinforcing particles did not deform plastically and remained in a significantly less worn state above the matrix surface, which resulted in less smooth and uniform surface topography compared with metallic coatings (Figure 9). The damage of the ceramic reinforcing particles observed in the SEM images included micro-cracks and chipping. The presence of the chromium carbides on the surface after the erosion tests and no visible voids left by fallen particles proves their excellent wetting and strong bonding with the matrix material, preventing their pull-out under the impact of the erodent. Since the Cr_3_C_2_ particles did not deform plastically, the erosion wear mechanism for the composite coatings is classified as mixed-mode: ductile for the matrix material and brittle for the reinforcing particles.

## 4. Conclusions

The laser cladding process was used to fabricate the composite Inconel 625 coatings reinforced with 20 and 40 vol.% of Cr_3_C_2_ particles. The effect of the process parameters and coatings compositions on the quality, macro- and microstructure, microhardness and solid particle erosion wear resistance was tested. The analysis of test results led to the following conclusions:

1. Laser cladding process is an effective method to fabricate defect-free metallic Inconel 625 and composite Inconel 625 coatings reinforced with 20–40 vol.% of Cr_3_C_2_, providing low dilution (below 10%). Laser beam power and linear energy input affect the dilution, thickness and risk of defects (lack of fusion and porosity) of the coatings. Increased laser beam power provided a slightly higher pore fraction; however, it reduced the risk of coating–substrate interface defects.
2. The metallic Inconel 625 coatings exhibit a typical dendritic microstructure consisting of an austenite matrix and minor constituents in the interdendritic regions. The grain size and morphology change with the distance from the coating–substrate interface due to changing local solidification conditions. The metallurgical bonding of the metallic coatings with the substrate is of high quality.
3. Cr_3_C_2_ particles are evenly distributed across the composite coatings and properly wetted by the liquid metal pool, resulting in high particle–matrix bond quality at all selected parameters. An increased fraction of the reinforcing particles led to a dilution decrease and a pore fraction increase. Cr_3_C_2_ particles promoted heterogeneous nucleation, leading to grain refinement of composite coatings compared with Inconel 625 coatings. Partial dissolution of Cr_3_C_2_ particles leads to the in situ formation of secondary Cr_7_C_3_ and Cr_23_C_6_ carbides, further modifying the matrix microstructure.
4. The reinforcement with 20–40 vol.% Cr_3_C_2_ particles results in a significant enhancement of the average microhardness by approximately 90% and 140%, respectively, compared with pure Inconel 625. The microhardness profiles are highly uniform throughout the coatings, confirming the high homogeneity of the laser-cladded coatings.
5. The addition of Cr_3_C_2_ particles improves the solid particle erosion resistance, particularly for the 30° impingement angle, where the erosion value decreased by 9% and 24% for the 20 and 40 vol.% Cr_3_C_2_ contents, respectively, compared with pure Inconel 625. The metallic coatings during solid particle erosion underwent plastic deformation, which confirms the ductile mechanism of erosive wear. In contrast, the composite coatings revealed the mixed-mode mechanism of erosive wear. While the matrix undergoes plastic deformation (ductile mechanism of erosive wear), the reinforcing particles exhibit a brittle mechanism of erosive wear. The absence of pull-out after erosion tests confirms the superior quality of the particle–matrix interface.

## Figures and Tables

**Table 1:** Chemical composition of S355JR steel substrate and Oerlikon Metcoclad 625 powder [24].

Material Designation	C	Mn	Si	P	S	Cr	Ni	Mo	Nb	Al	Cu	Fe
	wt.%											
S355JR	0.2	1.5	0.2–0.5	max 0.04	max 0.04	max 0.3	max 0.3	-	-	max 0.02	max 0.03	balance
Oerlikon Metcoclad 625	-	-	-	-	-	20.0–23.0	58.0–63.0	8.0–10.0	3.0–5.0	-	-	max 5.0

**Table 2:** Technical specifications of TRUMPF Trudisc 3302 laser [11].

Property	Value
Wavelength (μm)	1.3
Maximum output power (W)	3300
Laser beam divergence (mm∙rad)	<8.0
Fibre core diameter (μm)	200
Collimator focal length (mm)	200
Focusing lens focal length (mm)	200
Beam spot diameter (μm)	200
Fiber length (m)	20

**Table 3:** Laser cladding process parameters.

Designation	Cr_3_C_2_ Content (vol.%)	Laser Beam Power (W)	Speed (mm/min)	Powder Feed Rate (g/min)	Linear Energy Input (kJ/m)
I1	0	1300	200	8	390
I2	0	1600	200	8	480
C1-1	20	1300	200	8	390
C1-2	20	1600	200	8	480
C2-1	40	1300	200	8	390
C2-2	40	1600	200	8	480

**Table 4:** The coatings’ dilution and thickness results.

Designation (According to Table 3)	Dilution (%)	Thickness (mm)
I1	0.67 ± 0.1	1.5 ± 0.05
I2	4.41 ± 0.3	1.6 ± 0.04
C1-1	6.53 ± 0.6	0.9 ± 0.04
C1-2	10.06 ± 1.1	1.1 ± 0.06
C2-1	0.88 ± 0.2	1.4 ± 0.03
C2-2	2.59 ± 0.5	1.6 ± 0.05

**Table 5:** Average microhardness results.

Designation (According to Table 3)	Average Microhardness (HV0.2)
I1	225 ± 8.9
I2	222 ± 11.6
C1-1	427 ± 64.7
C1-2	392 ± 26.2
C2-1	492 ± 53.0
C2-2	533 ± 41.2

**Table 6:** Average solid particle erosion results.

Designation (According to Table 3)	Steady-State Erosion Rate (mg/min)		Erosion Value (mm^3^/g)	
	Impingement Angle			
	30°	90°	30°	90°
I2	0.15 ± 0.02	0.09 ± 0.01	0.009 ± 0.0009	0.0051 ± 0.0007
C1-2	0.13 ± 0.02	0.08 ± 0.01	0.0082 ± 0.0013	0.0051 ± 0.0009
C2-2	0.11 ± 0.02	0.08 ± 0.02	0.0068 ± 0.001	0.0051 ± 0.0012

**Table 7:** Two-way ANOVA results of the steady-state erosion rate and erosion value between the coating type and impingement angle.

Source	*p* Value	
	Steady-State Erosion Rate	Erosion Value
Coating	0.043	0.204
Impingement angle	0.000046	0.000055
Coating × impingement angle	0.151	0.209

## Data Availability

The original contributions presented in the study are included in the article, and further inquiries can be directed to the corresponding authors.

## Abbreviations

The following abbreviations are used in this manuscript:

MMC	Metal Matrix Composite
HVOF	High Velocity Oxygen Fuel Spraying
EDS	Energy Dispersive Spectroscopy
XRD	X-Ray Diffraction
SEM	Scanning Electron Microscope
HAZ	Heat-Affected Zone
